# Extracellular matrix stiffness determines the phenotypic behavior of dedifferentiated melanoma cells through a DDR1/2-dependent YAP mechanotransduction pathway

**DOI:** 10.1038/s41389-026-00636-y

**Published:** 2026-06-13

**Authors:** Margaux Lecacheur, Ilona Berestjuk, Alexandrine Carminati, Mira Rabbaa, Océane Bouvet, Serena Diazzi, Pierric Biber, Christopher Rovera, Marie Irondelle, Frédéric Larbret, Virginie Prod’homme, Christophe A. Girard, Marcel Deckert, Sophie Tartare-Deckert

**Affiliations:** 1 Université Côte d’Azur, INSERM, C3M Nice, France; 2 Team “Microenvironment, Signaling and Cancer”, Equipe labellisée Ligue Contre le Cancer, Nice, France; 3 Université Côte d’Azur, INSERM, C3M, Microscopy Facility, Nice, France

**Keywords:** Cancer microenvironment, Cell signalling

## Abstract

Extracellular matrix (ECM) stiffening is a biophysical hallmark of solid tumors. Cutaneous melanoma is an aggressive malignancy characterized by high heterogeneity and phenotypic plasticity in which melanoma cells switch from a proliferative and differentiated phenotype to an invasive, dedifferentiated and therapy-resistant state. However, the impact of ECM stiffness on the diverse cellular phenotypes of melanoma remains poorly defined. Here, we show that melanoma cell subpopulations differ in their responses to mechanical signals. Compared to melanocytic/transitory cells, dedifferentiated cells exhibited heightened sensitivity to stiff collagen matrices, characterized by increased cell spreading, focal adhesion maturation, YAP nuclear translocation and contractility. ECM stiffening enhanced proliferation, migration and invasion in dedifferentiated cells, whereas highly proliferative and poorly migratory melanocytic/transitory cells were less affected by collagen stiffness. Importantly, a soft ECM sensitized dedifferentiated cells, but not melanocytic/transitory cells, to BRAF/MEK inhibition. Mechanistically, the mechanosensitivity of dedifferentiated cells relies on collagen receptors DDR1 and DDR2, which control cytoskeleton reorganization and YAP mechanosignaling. Genetic or pharmacological inhibition of DDR, actomyosin contractility, or YAP suppressed stiffness-induced proliferation and migration, reduced traction forces, and restored sensitivity to targeted therapy in dedifferentiated cells. Conversely, ectopic DDR1/DDR2 expression confers mechanosensitive properties to melanocytic cells. Our results thus reveal that phenotypic plasticity endows dedifferentiated melanoma cells with increased addiction to mechanical cues and implicate DDR1/2-YAP-dependent signaling in this aggressive behavior.

**Figure:**
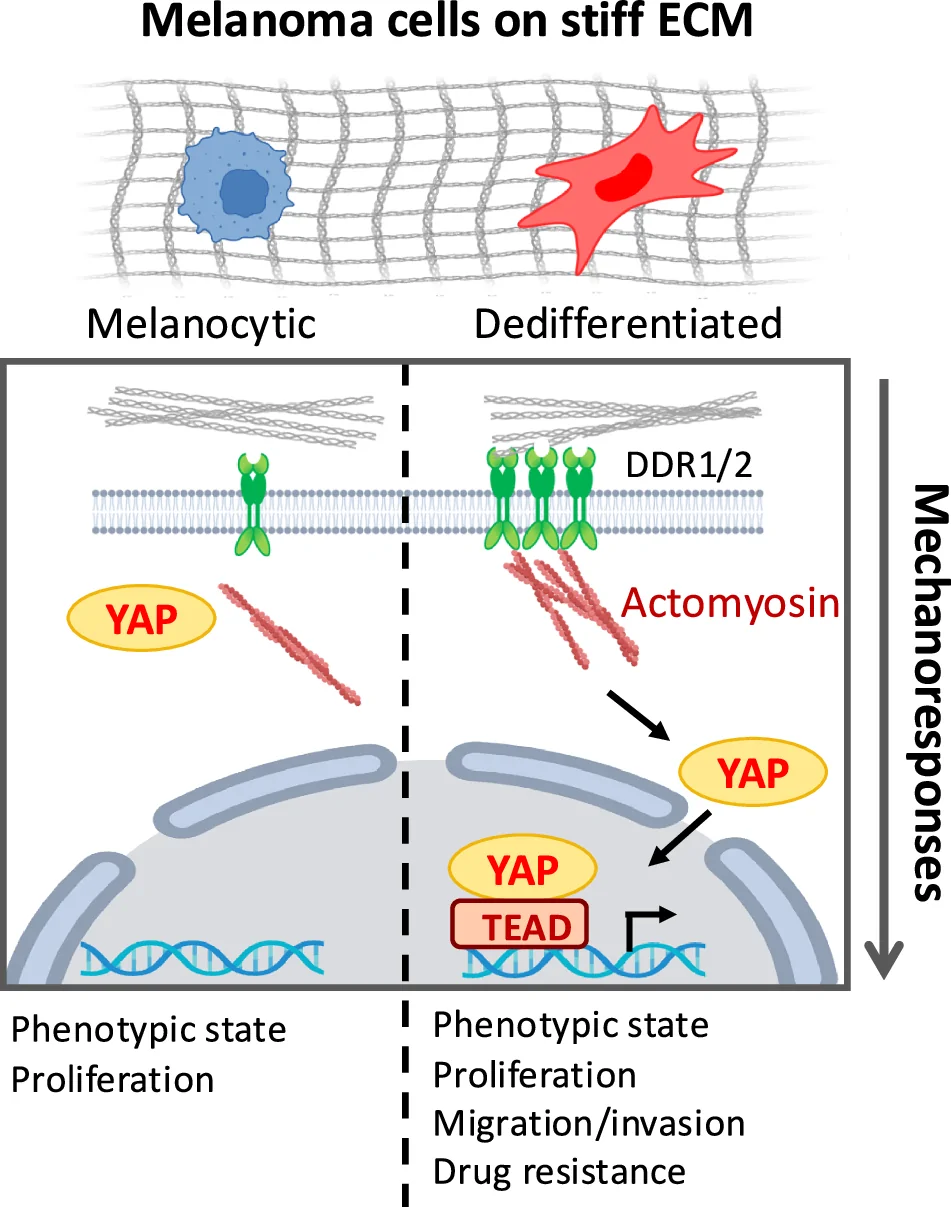

## Introduction

Extracellular matrix (ECM) stiffness-induced signaling pathways play a crucial role in cell behavior and tissue homeostasis [1]. The ECM, a complex and dynamic network of proteins, provides structural support to surrounding cells and regulates cellular functions [2]. Cells sense and respond to ECM mechanical cues through ECM receptors and signaling pathways that regulate gene expression [3]. These pathways include integrin-mediated focal adhesion kinase (FAK) activation, Rho/ROCK-actomyosin signaling, and the Hippo-YAP/TAZ pathway [4, 5]. While the role of β1-integrin in mechanotransduction has been well described, the contribution of other ECM receptors such as the discoidin domain tyrosine kinase receptors DDR1 and DDR2 is less well appreciated. In the context of cancer, altered ECM deposition and increased collagen cross-linking leading to tumor stiffening is known to foster the growth, progression and recurrence of many solid tumors [1].

Melanoma is the primary cause of skin cancer-related deaths due to its high intra-tumor heterogeneity and plasticity leading to therapy failure and metastatic propensity. Most mutations found in cutaneous melanoma affect key components of the MAP kinase signaling pathway, including *BRAF* (40–50%), *NRAS* (20–30%), and *NF1* (10–15%) resulting in its constitutive activation [6]. BRAF-mutated metastatic melanomas are treated with targeted therapy combining BRAF and MEK inhibitors (BRAFi/MEKi) or immune checkpoint inhibitors [7]. While both treatments have improved the clinical outcomes of metastatic melanoma, not all patients respond to these regimens, and those who initially respond eventually relapse due to the development of resistance.

Melanoma exhibits a high degree of phenotypic and behavioral heterogeneity, which results from the ability of melanoma cells to dynamically oscillate between distinct transcriptomic and phenotypic states. This plasticity enables them to rapidly adapt to external cues and stressful conditions. The most identified melanoma cell states are the melanocytic differentiated phenotype, and the mesenchymal-like dedifferentiated phenotype [8]. Melanocytic cells are proliferative and drug sensitive. They express high levels of melanocyte lineage-specific microphtalmia-associated transcription factor (MITF) and low levels of the receptor tyrosine kinase AXL. In contrast, dedifferentiated cells are poorly proliferative, highly invasive and therapy resistant. They exhibit low MITF expression and high expression of mesenchymal markers including AXL and ZEB1 [9, 10]. The transition from a melanocytic to a dedifferentiated phenotype occurs through multiple intermediate cell states including a transient neural crest stem cell like (NCSC) population, which contributes to the development of non-genetic resistance [11].

Phenotype switching in melanoma is triggered by extracellular cues, including soluble factors (i.e., TGFβ, INFγ), nutrient deprivation, hypoxia or oncogenic BRAF-targeting therapy [12–17]. Besides phenotypic switching, targeted therapy promotes stromal and melanoma cell-derived ECM deposition and remodeling, leading to tumor stiffening and drug relapse [18–22]. Notably, biophysical and structural cues from the ECM confer drug-protective action to melanoma cells against anti-BRAF/MEK therapies [19, 23, 24]. Collagen remodeling is also observed in aging skin and promotes melanoma metastasis [25]. In epithelial cancers, increased ECM stiffness promotes metastasis through the induction of epithelial to mesenchymal transition (EMT), a transdifferentiation process resembling melanoma mesenchymal-like transition [26, 27]. Recently, collagen abundance has been shown to promote melanoma cell differentiation, suggesting that ECM rigidity may regulate phenotypic plasticity [28]. However, it remains to be determined whether ECM stiffness differently impacts on the various melanoma cell states to drive malignant features and aggressiveness.

Here we sought to investigate how mechanical signals from the ECM influence melanoma cell phenotypic behaviors. We selected a panel of melanoma cell lines and patient-derived melanoma short-term cultures presenting either a melanocytic/transitory or dedifferentiated phenotype and exposed them to collagen matrices of increasing stiffness. We provide evidence that lineage dedifferentiation increases sensitivity and responses to stiff ECM and identify the collagen receptors DDR1 and DDR2 as key players in mediating mechano-responses in dedifferentiated cells. Our findings highlight a novel vulnerability of dedifferentiated melanoma cells on mechanical signals that could be potentially exploited to target this aggressive, treatment-refractory tumor cell state.

## Results

### Dedifferentiated melanoma cells are more sensitive to ECM stiffening than melanocytic cells

**Fig. 1:**
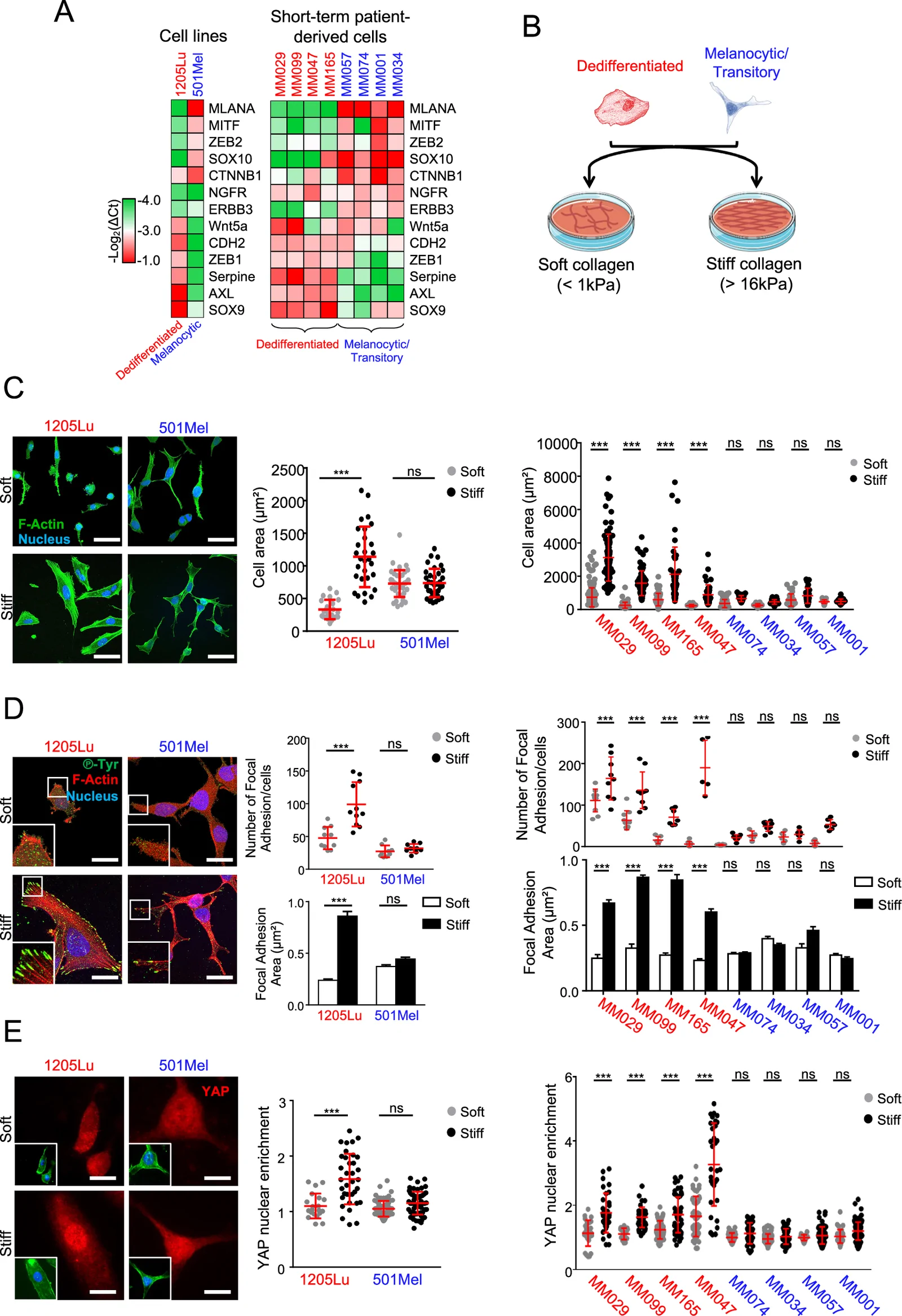
Dedifferentiated melanoma cells display mechanical responsiveness compared to melanocytic cells. **A** Expression heatmap of phenotype switching markers in melanoma cell lines (*left*) and short-term patient derived melanoma cells (*right*) used in the study. Dedifferentiated cells are in red and melanocytic/transitory cells in blue. **B** Cartoon illustrating the experimental design used to assess melanoma cell sensitivity to collagen-coated substrates of increasing stiffness. **C** *Left*, representative images of the morphology of dedifferentiated (1205Lu) or melanocytic (501Mel) melanoma cells cultured for 72 h on soft versus stiff collagen-coated substrates. F-Actin was stained with phalloidin (green) and nuclei with Hoechst (blue). Scale bar, 50 µm. *Right*, quantification of morphological changes of melanoma cell lines (*left*) or short-term patient derived melanoma cells (*right*) cultured as above. Data are representative of 3 independent experiments (*n* > 150 cells). Data are represented as scatter plot with mean ± SD. ***P < 0.001; ns, non-significant, Kruskal-Wallis analysis. **D** *Left*, immunofluorescence analysis of focal adhesions of 1205Lu or 501Mel cells cultured for 72 h on collagen-coated soft or stiff substrate. Focal adhesions were stained with anti-Phospho-Tyrosine antibody (green), F-Actin with phalloidin (red) and nuclei with Hoechst (blue). Insets show higher magnification views of delineated zones. Scale bar, 25 µm. *Right-top*, number of focal adhesions per cell of melanoma cell lines (*left*) or short-term patient derived melanoma cells (*right*). Data are representative of 3 independent experiments. Each dot represents a field with 3–10 cells. (*n* = 10 fields). Data are represented as scatter plot with mean ± SD. ***P < 0.001; ns, non-significant, Kruskal-Wallis analysis. *Right-bottom*, bar graphs show the focal adhesion area in cell lines (*left*) or short-term patient derived cells (*right*). Data represent the mean ± SD (*n* > 150 cells) of triplicate experiments. ***P < 0.001; ns, non-significant, Kruskal-Wallis analysis. **E** *Left*, effect of collagen stiffening on YAP nuclear translocation assessed by immunofluorescence in cells cultured for 72 h on soft or stiff collagen substrates. Insets show F-Actin in green and nuclei in blue. Scale bar, 15 µm. *Right*, YAP nuclear enrichment is represented as scatter plot with mean ± SD (*n* ≥ 30 cells per condition). Data are representative of 3 independent experiments. ***P < 0.001; ns non-significant, Kruskal-Wallis analysis.

Melanoma cells are sensitive to ECM stiffening [19, 28] yet whether their mutation status or transcriptional state influences responsiveness to substrate rigidity is unknown. To test this, we selected melanoma cell lines and short-term melanoma cultures (designated as MM) harboring either a BRAF or NRAS mutation. qPCR analysis of melanoma phenotype switching genes confirmed that the melanocytic/transitory phenotype of 501Mel, MM057, MM074, MM034, MM001 and MM057 cells is associated with high expression levels of *MITF*, *SOX10* or *MLANA*. In contrast, 1205Lu, MM029, MM099, MM047, and MM165 cells exhibit a MITF^low^/AX^high^ dedifferentiated signature associated with the increased expression of *AXL*, *SOX9* and *ZEB1* (Supplementary Table 1, Fig. 1A). To assess their sensitivity to changes in ECM stiffness conditions, cells were plated on collagen-coated polyacrylamide hydrogels of low ( < 1 kPa) versus high ( > 16 kPa) stiffness (Fig. 1B). Culturing dedifferentiated 1205Lu cells for 72 h on stiff collagen induced an increase in their spreading and cell area, while it had no significant effect on melanocytic 501Mel cells (Fig. 1C). Compared to 501Mel cells, 1205Lu cells formed mature focal adhesions upon increased ECM rigidity (Fig. 1D). Similar observations were made with melanocytic/transitory versus dedifferentiated MM cells (Fig. 1C, D and Supplementary Fig. 1A, B). In addition, dedifferentiated melanoma cells showed activation of YAP-dependent mechanosensing pathway, illustrated by YAP nuclear translocation and increased expression of its target genes (*AMOTL2*, *CTGF*, *CYR61*) under stiff collagen (Fig. 1E and Supplementary Fig. 1C, E). Interestingly, both BRAF-mutated (1205Lu, MM029, MM099) and NRAS-mutated (MM047, MM165) dedifferentiated melanoma cells were much more sensitive to collagen stiffness than BRAF- or NRAS-mutated melanocytic cells. Analysis of a publicly available melanoma cell gene expression dataset [17] revealed elevated expression of *YAP* and its target gene *CYR61* in dedifferentiated (undifferentiated and neural crest–like) cell lines compared with melanocytic cell lines. This finding is consistent with the increased nuclear localization and activity of YAP observed in dedifferentiated cells cultured on stiff collagen. *AXL* and *MITF* served as markers of the dedifferentiated and melanocytic states, respectively (Supplementary Fig. 1F). Overall, these data indicate that melanoma cells presenting a dedifferentiated phenotype are more sensitive to extracellular mechanical signals than melanocytic/transitory melanoma cells, regardless of their mutation status.

### ECM stiffening increases the proliferation of dedifferentiated melanoma cells and their resistance to BRAF mutant-targeting therapy

**Fig. 2:**
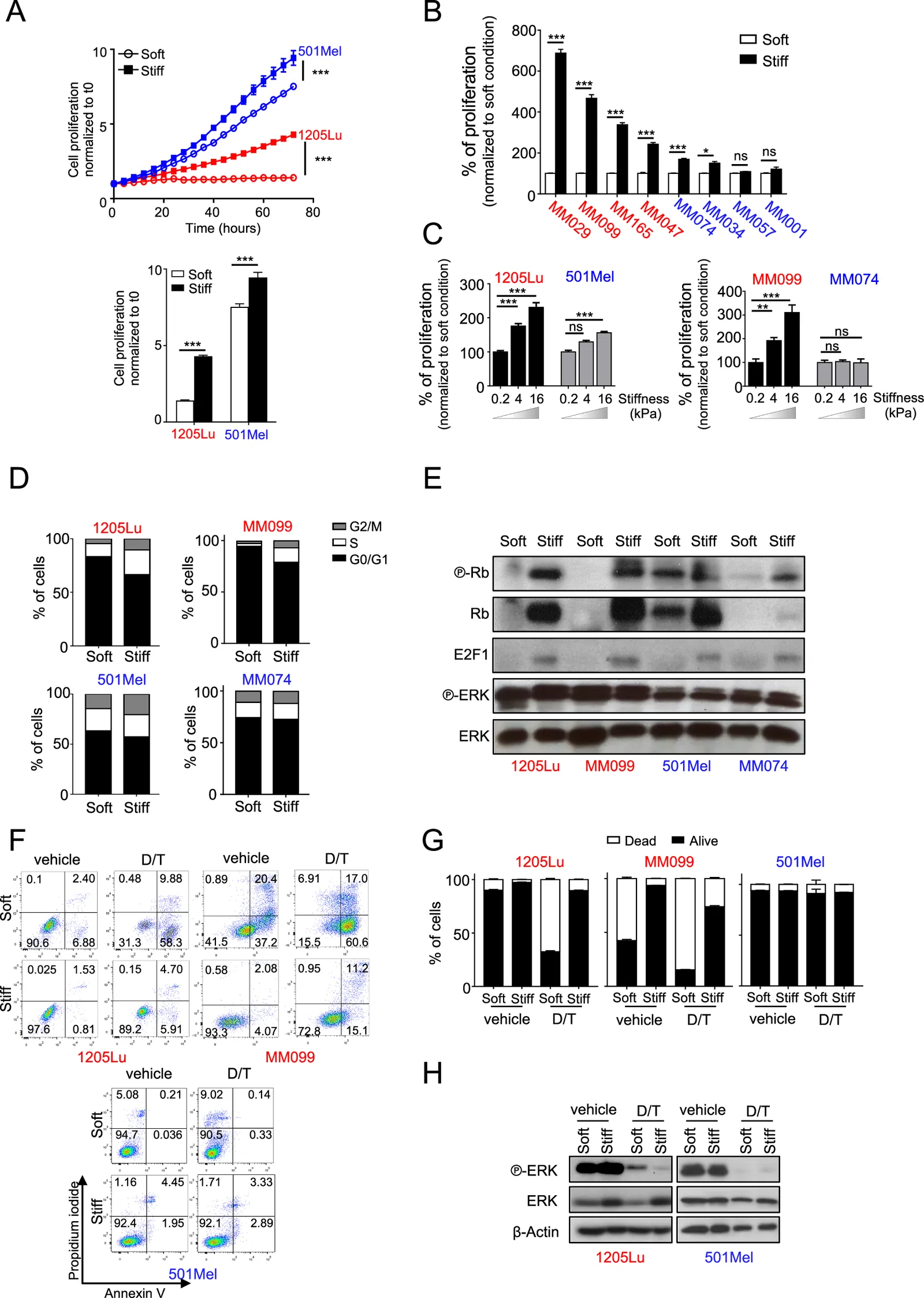
Dedifferentiated melanoma cells display mechano-dependent proliferative and drug resistance properties. **A** *Top*, time lapse analysis of 501Mel or 1205Lu melanoma cell lines proliferation cultured for 72 h on soft or stiff collagen-coated gels, analyzed using the IncuCyte system. Graphs show quantification of cell numbers from NucLight Red nuclear object counting. Data are the mean ± SD, from a minimum of 10 fields per conditions. ***P < 0.001, two-way ANOVA analysis. *Bottom*, bar graph of cell proliferation after 72 h culture on soft or stiff collagen-coated substrate. Data are normalized to T0. ***P < 0.001, Kruskal-Wallis analysis (*n* = 3). **B** Bar graph of short-term patient-derived melanoma cell proliferation measured at 72 h post seeding on soft or stiff collagen-coated substrate. Data are normalized to proliferation on soft condition ***P < 0.001, Kruskal-Wallis analysis; ns, non-significant, Kruskal-Wallis analysis. Data are representative of 3 independent experiments. **C** Bar graph of proliferation of 1205Lu versus 501Mel cell lines (*left*) or MM099 versus MM074 short-term patient derived cells (*right*) seeded on polyacrylamide collagen-coated hydrogels of increasing stiffness. Data are normalized to proliferation on soft condition. Data are representative of 3 independent experiments. ***P < 0.001; ns, non-significant, Kruskal-Wallis analysis. **D** Cell cycle distribution of melanoma cell lines (1205Lu or 501Mel) or short-term patient-derived melanoma cells (MM099 or MM074) plated for 72 h on the substrates as in (**A**). **E** Immunoblot analysis of cell cycle markers in lysates from dedifferentiated (1205Lu and MM099) and melanocytic/transitory (501Mel and MM074) cells from (**D**). ERK is used as loading control. **F** Quantification of apoptosis in 1205Lu, MM099 or 501Mel cells seeded on soft or stiff collagen-coated substrate and exposed for 72 h to vehicle or 1 µM of Dabrafenib and 0.1 µM of Trametinib (D/T). The percentage of apoptotic cells was determined by flow cytometry analysis of annexin V-FITC/PI-stained cells. Early and late apoptotic cells are shown in the right lower quadrant and right upper quadrant, respectively. **G** Bar graph showing the percentage of dead (*white*) and live (*black*) cells from **F**. Dead cells (necrotic and apoptotic cells) are shown in the right lower quadrant, right and left upper quadrants. Data represent the mean ± SD of triplicate experiments. **H** Immunoblot analysis of ERK1/2 phosphorylation in lysates from 1205Lu or 501Mel melanoma cell lines obtained from (**F**). β-Actin is used as loading control.

ECM-derived mechanical signals have been shown to increase the growth of various solid cancers and to decrease their response to anti-cancer treatments [1, 19, 20, 24]. We therefore examined the functional consequences of increased stiffness on melanoma cell subpopulations. First, we compared the proliferation of melanocytic and dedifferentiated melanoma cells cultured at increasing levels of collagen stiffness using live cell imaging. As predicted, melanocytic 501Mel cells were more proliferative that dedifferentiated invasive 1205Lu cells and less affected by collagen stiffness (Fig. 2A). Most melanoma cells studied showed enhanced proliferation on stiff collagen, which was stronger with dedifferentiated cells when compared to melanocytic/transitory cells, with no detectable effect observed for MM057 and MM001 cells (Fig. 2B, C). Comparable results were obtained using a Ki67 proliferation marker staining (Supplementary Fig. 2A, B). Remarkably, the proliferation of dedifferentiated melanoma cells was blocked on soft substrate (Fig. 2A). Cell cycle analysis showed that low stiffness induced a G0/G1 cell cycle arrest in dedifferentiated melanoma cells whereas it only had a modest effect in differentiated ones (Fig. 2D and Supplementary Fig. 2D). At the molecular level, a strong reduction in the expression of proliferation markers P-Rb and E2F1 was observed in 1205Lu and MM099 dedifferentiated cells (Fig. 2E). Finally, analysis of protein levels of four differentiation state markers (MITF, SOX10, AXL and ZEB1) showed stiffness-dependent expression of phenotypic markers in both melanocytic/transitory and dedifferentiated cells (Supplementary Fig. 2C). Altogether, these results suggest that the phenotypic molecular pattern and the proliferation of dedifferentiated melanoma cell are dependent on ECM stiffness.

We next wondered whether the increased mechanosensitivity of dedifferentiated melanoma cells influences their response to oncogenic BRAF inhibition. We seeded dedifferentiated or melanocytic/transitory cells on soft or stiff collagen and exposed them for 72 h to the therapy targeting mutant BRAF (BRAFi, Dabrafenib) and MEK (MEKi, Trametinib). On stiff substrate, Dabrafenib/Trametinib (D/T) combination induced a G0/G1 cell cycle arrest in both melanocytic and dedifferentiated cells (Supplementary Fig. 2E). However, on soft substrate D/T increased the number of apoptotic cells in 1205Lu and MM099 dedifferentiated melanoma cells, but not in 501Mel melanocytic cells (Fig. 2F, G). As control, phosphorylation of ERK/MAP kinase was strongly reduced upon administration of the drugs in soft and stiff conditions (Fig. 2H). These data suggest that low collagen stiffness significantly increases sensitivity to BRAFi/MEKi combination and, conversely, that ECM stiffening confers drug resistance to dedifferentiated melanoma cells. Further, they also indicate that the response of melanocytic differentiated cells to targeted therapy is unaffected by ECM stiffness.

### ECM stiffening promotes the migratory and invasive behavior of dedifferentiated melanoma cells

**Fig. 3:**
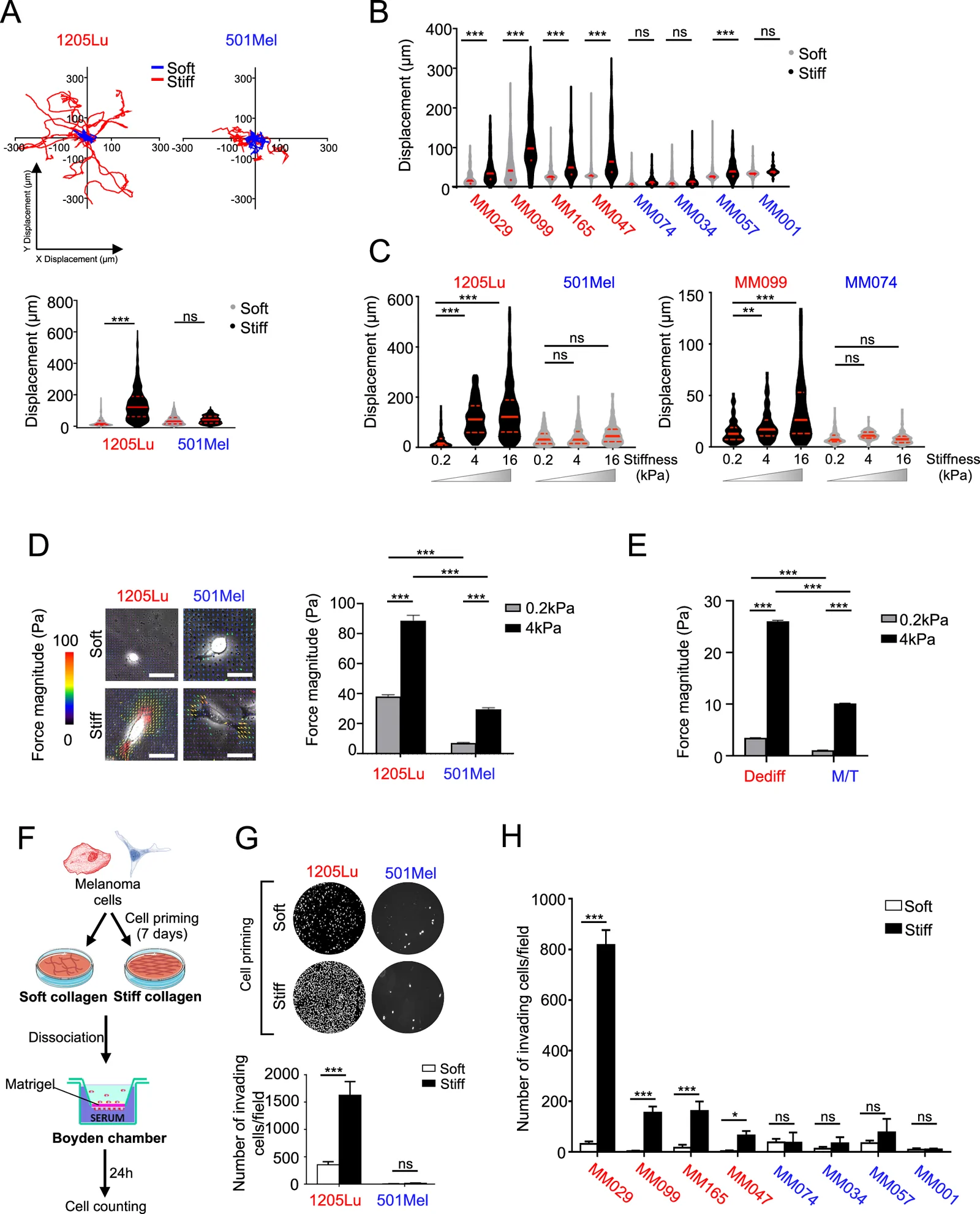
Collagen stiffening increases dedifferentiated melanoma cell motile and invasive behavior. **A** *Top*, dedifferentiated (1205Lu) or melanocytic (501Mel) melanoma cells were seeded on soft or stiff collagen-coated substrates for 24 h, and cell migration was recorded over 24 h and automatically tracked using Image J. For each condition, spider plots were created and cell displacement was analyzed using the ImageJ. *Bottom*, quantification of cell displacement (n = 200 cells). Data represent median ± quartiles. ***P < 0.001; ns non-significant, Kruskal-Wallis analysis. **B** Violin plot representing cell displacement of short-term patient derived cells seeded as in (**A**). Data represent median ± quartiles. ***P < 0.001; ns, non-significant, Kruskal-Wallis analysis. **C** Quantification of cell displacement of indicated melanoma cells seeded on collagen-coated polyacrylamide hydrogels of increasing stiffness for 24 h (*n* < 300 cells). Data represent median ± quartiles. Data are representative of 3 independent experiments. ***P < 0.001; **P < 0.01; ns non-significant, Kruskal-Wallis analysis. **D** *Top*, representative images of heat scale plot showing the traction forces applied by 1205Lu or 501Mel cells seeded on 0.2 or 4 kPa fluorescent bead-embedded collagen coated hydrogels for 24 h. Scale bar, 75 µm. *Bottom*, Bar graph showing the quantification of contractile forces. Data are the mean ± SD (*n* = 10 cells/field from 5 random fields). Data are representative of 3 independent experiments. ***P < 0.001; ns non-significant, Kruskal-Wallis analysis. **E** Quantitative analysis of contractile forces applied by short-term patient derived melanoma cells grouped as dedifferentiated (Dediff) (MM029, MM099, MM165, MM047) or melanocytic/transitory (M/T) (MM074, MM034, MM057, MM001) on 0.2 or 4 kPa fluorescent bead-embedded collagen coated hydrogels for 24 h. Data are represented as mean ± SD of 3 independent experiments. ****P < 0.0001, Kruskal-Wallis analysis. **F** Dedifferentiated or melanocytic melanoma cells were seeded for 7 days on soft or stiff collagen-coated substrates, then detached and counted. Their invasive properties were subsequently analyzed in Boyden chamber for 24 h. **G** Representative images (*top*) and quantification (*bottom*) of invading cells in the different conditions. Bar graph represents the mean ± SD of 3 independent experiments. ***P < 0.001; ns, non-significant, Kruskal-Wallis analysis. **H** Quantification of invasive properties of indicated short-term patient derived cells treated as in (**F**). Data represent the mean ± SD of 3 independent experiments. ***P < 0.001; *P < 0.05; ns non-significant, Kruskal-Wallis analysis.

We next assessed the effect of ECM stiffness on motility and invasiveness of melanoma cells. First, we seeded 501Mel melanocytic or 1205Lu dedifferentiated cells on collagen substrates with increasing stiffness and monitored individual cell motility using live cell imaging. Neither 501Mel nor 1205Lu cells migrated when cultured on soft collagen substrate. However, plating cells on stiff collagen induced a higher increase in 1205Lu cell motility than in 501Mel (Fig. 3A, C). Similar results were obtained in short-term melanoma cells (Fig. 3B, C). As cell migration depends on the ability of cells to contract their actomyosin cytoskeleton and the surrounding substrate, we compared traction stresses generated by melanoma cell lines or short-term cultures using TFM. We observed that dedifferentiated cells applied stronger forces on collagen-coated stiff matrices than melanocytic/transitory cells (Fig. 3D, E). Finally, we questioned whether priming melanoma cells on different rigidity would affect their invasive properties. To do so, we cultured melanoma cells on soft or stiff collagen substrates for a week, dissociated them and assessed their invasion ability using transwell chambers coated with matrigel (Fig. 3F). We found that pre-culturing dedifferentiated 1205Lu cells on stiff substrate increased their invasion ability (Fig. 3G). In contrast, priming melanocytic 501Mel cells on stiff substrate did not affect their invasion ability. Similar observations were obtained with short-term melanoma cells (Fig. 3H). Together these results suggest that a stiff ECM regulates the migratory and invasive phenotypes of dedifferentiated melanoma cells.

### Collagen receptors DDR1 and DDR2 mediate melanoma cell mechano-responsiveness

**Fig. 4:**
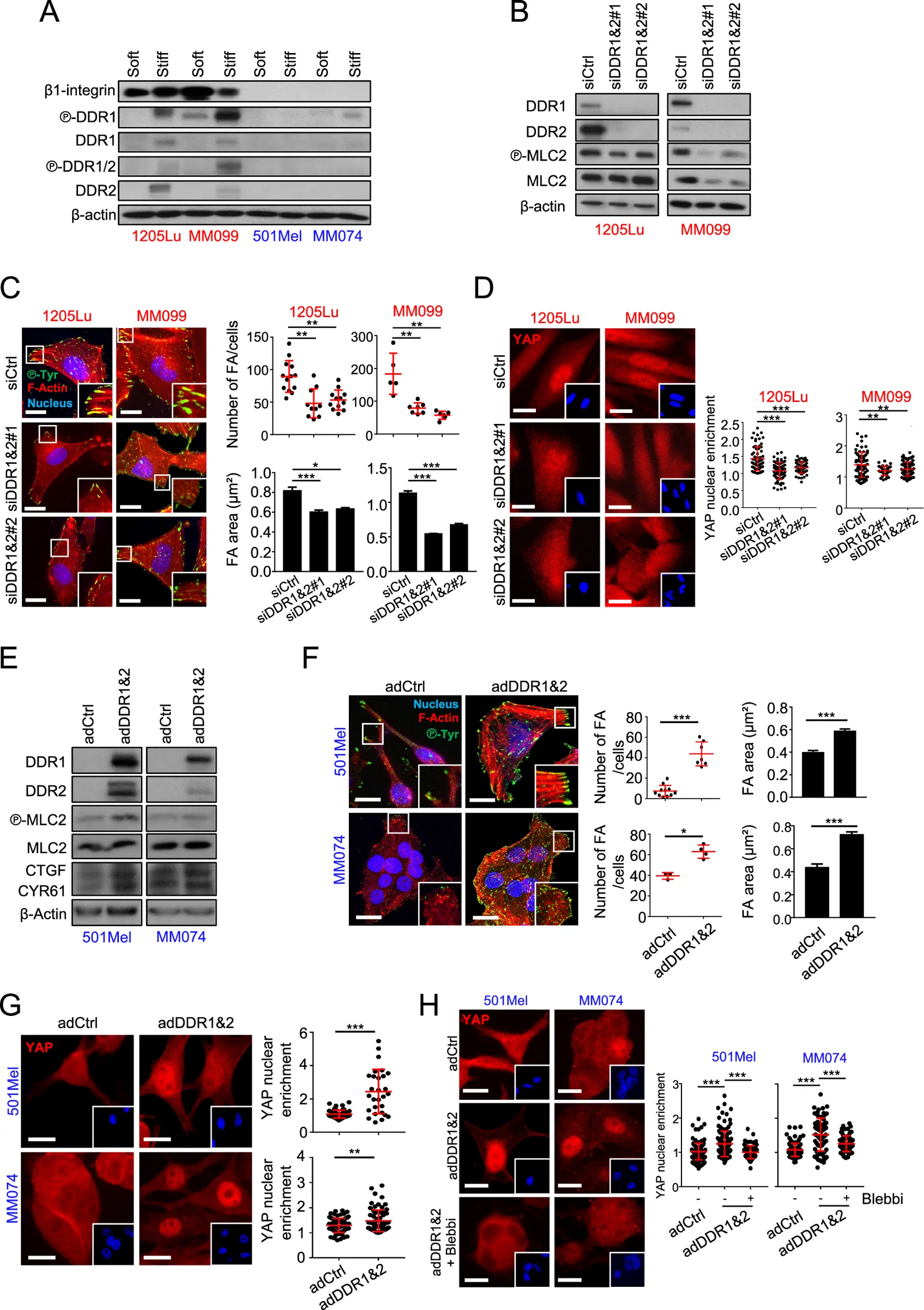
Collagen receptors DDR1 and DDR2 mediate melanoma cell mechano-responsiveness. **A** Immunoblot analysis of β1-integrin, DDR1 and DDR2 in lysates from dedifferentiated or melanocytic melanoma cells cultured for 72 h on soft or stiff collagen-coated substrate. β-Actin is used as loading control. **B**–**D** 1205Lu or MM099 dedifferentiated cells on stiff collagen substrate were transfected with the combination of two different sequences of siRNA targeting DDR1 or DDR2 (siDDR1&2#1 and siDDR1&2#2) or control siRNA (siCtrl) (72 h, 50 nM). **B** Immunoblot analysis of DDR1, DDR2 and MLC2 in lysates from 1205Lu or MM099 cells 72 h post transfection with the indicated siRNA. β-Actin is used as loading control. **C** *Left*, immunofluorescence analysis of focal adhesions (FA) of 1205Lu or MM099 cells described above. Focal adhesions were stained with anti-Phospho-Tyrosine antibody (green), F-Actin with phalloidin (red) and nuclei with Hoechst (blue). Insets show higher magnification views of zones delineated by a white square. Scale bar, 20 µm. *Right*, quantification of FA per cell (*top*) or FA area (*bottom*). Data are representative of 3 independent experiments. *Top*, each dot represents a field comprising 3–10 cells. Data are represented as scatter plot with mean ± SD from 10 different fields. ***P < 0.001; ns, non-significant, Kruskal-Wallis analysis. *Bottom*, Bar graph represents the mean ± SD (*n* > 150 cells) of triplicate experiments. ***P < 0.001; ns, non-significant, Kruskal-Wallis analysis. **D** *Left*, YAP nuclear translocation assessed by immunofluorescence in 1205Lu or MM099 cells described above. Insets show nuclei staining by Hoechst (blue). Scale bar, 20 µm. *Right*, quantification of the nucleocytoplasmic distribution of YAP (*n* ≥ 30 cells per condition). Data represented mean ± SD of 3 independent experiments. ***P < 0.001; ns, non-significant, Kruskal-Wallis analysis. **E**–**H** 501Mel or MM074 cells seeded on stiff collagen substrate were transduced with the combination of adenoviruses expressing DDR1 or DDR2 (adDDR1&2) or a control adenovirus (adCtrl). **E** Immunoblot analysis of DDR1, DDR2, MLC2 and YAP target genes (CTGF and CYR61). β-Actin is used as loading control. **F** *Left*, immunofluorescence analysis of focal adhesions (FA) of 501Mel or MM074 cells treated as above. FA were stained with anti-Phospho-Tyrosine antibody (green), F-Actin with phalloidin (red) and nuclei with Hoechst (blue). Insets show higher magnification views of zones delineated by a white square. Scale bar, 20 µm. *Right*, quantification of FA per cell (*top*) or FA area (*bottom*). Data are representative of 3 independent experiments. *Top*, data are represented as scatter plot with mean ± SD from 10 different fields. Each dot represents a field comprising 3–10 cells. ***P < 0.001; ns, non-significant, Kruskal-Wallis analysis. *Bottom*, bar graph represents the mean ± SD (*n* > 150 cells) of triplicate experiments. ***P < 0.001; ns, non-significant, Kruskal-Wallis analysis. **G** *Left*, immunofluorescence analysis of YAP (red) in 501Mel and MM074 cells. Insets show nuclei staining by Hoechst. Scale bar, 20 µm. *Right*, quantification of the nucleocytoplasmic distribution of YAP (*n* ≥ 30 cells per condition). Data represented mean ± SD of 3 independent experiments. ***P < 0.001; ns, non-significant, Kruskal-Wallis analysis. **H** *Left*, representative Images of YAP staining in 501Mel and MM074 cells seeded on stiff substrate and overexpressing DDR1 and DDR2. Cells were treated or not with the myosin II inhibitor Blebbistatin (Blebbi, 40 µM) along with adenovirus for 72 h. Insets represent nuclei stained with Hoechst. Scale bar, 20 µm. *Right*, Quantification of the nucleocytoplasmic distribution of YAP (*n* ≥ 30 cells per condition). Data represent mean ± SD. Data are representative of 3 independent experiments. ***P < 0.001; ns, non-significant, Kruskal-Wallis analysis.

Previous studies have shown that members of the integrin family and DDR receptors sense the physical properties of the ECM and transduce mechanical forces to cellular responses [24, 29–31]. β1-integrin was overexpressed in dedifferentiated melanoma cells relative to melanocytic/transitory states, but its response to matrix stiffness varied across cell lines. By contrast, DDR1 and DDR2 expression and phosphorylation were more consistently upregulated by collagen stiffness (Fig. 4A). These observations prompted us to investigate the role of DDR1 and DDR2 in melanoma cell responses to mechanical cues. To address this, we either used a siRNA approach to downregulate their expression (Fig. 4B) or a pharmacological approach to inhibit their tyrosine kinase enzymatic activity in dedifferentiated cells (Supplementary Fig. 3A). Immunoblot analysis showed that both approaches led to a decrease in phosphorylation of myosin light chain 2 (MLC2), the regulatory subunit of myosin II and key regulator of actomyosin contractility. In addition, the simultaneous knockdown of both DDR or their pharmacological inhibition in 1205Lu and MM099 cells plated on stiff substrate led to a decrease in the number and size of focal adhesions (Fig. 4C and Supplementary Fig. 3B), a significant reduction of YAP nuclear localization (Fig. 4D and Supplementary Fig. 3C) as well as its transcriptional activity (Supplementary Fig. 3D). These data indicates that DDR1 and DDR2 are required to mediate ECM mechanical signals in dedifferentiated melanoma cells. Finally, analysis of publicly available gene expression networks from the TCGA showed a positive correlation between *DDR1*, *DDR2* and *YAP* expression in human melanoma biopsies (Supplementary Fig. 3E) and a correlation between high levels of *DDR1/DDR2/YAP* expression and reduced melanoma patient survival (Supplementary Fig. 3F), emphasizing the close link between YAP and DDR.

We next asked whether DDR would be sufficient to activate mechanosensitive cell responses. To address this, we overexpressed both receptors in melanocytic melanoma cells using an adenoviral approach. Interestingly, forcing DDR1 and DDR2 expression in 501Mel or MM074 cells led to an increased phosphorylation of MLC2 (Fig. 4E). This was accompanied by the acquisition of a less differentiated phenotype, as shown by the loss of MITF expression and the increase in ZEB1 expression (Supplementary Fig. 3G). Compared to control cells, DDR-overexpressing melanocytic/transitory cells, plated on stiff collagen substrate, appeared more spread out (Supplementary Fig. 3H), formed more mature focal adhesions (Fig. 4F), and displayed increased nuclear localization and activation of YAP (Fig. 4E, G and Supplementary Fig. 3I) along with no significant effect on cell proliferation (Supplementary Fig. 3J). Notably, targeting myosin II with Blebbistatin reversed DDR-induced YAP nuclear activation (Fig. 4H). Collectively, these experiments indicate that DDR1 and DDR2 control mechanosensitive properties of melanoma cells through the actomyosin-dependent regulation of YAP pathway.

### Targeting DDR, actomyosin or YAP impairs stiff collagen-mediated dedifferentiated melanoma cell phenotypic behavior

**Fig. 5:**
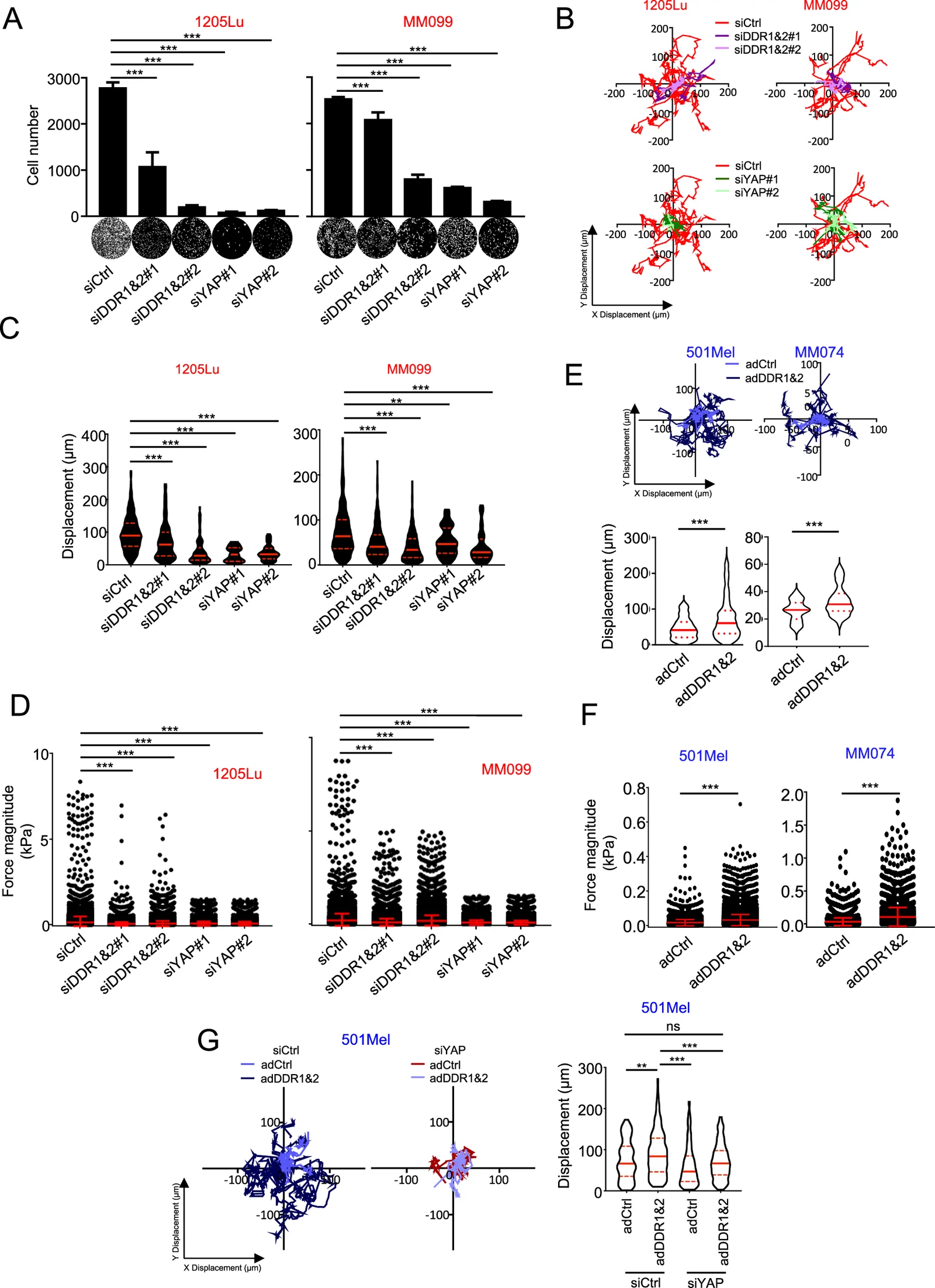
Targeting DDR or YAP impairs ECM stiffness-mediated proliferation and migration in dedifferentiated melanoma cells. **A** Proliferation analysis of 1205Lu and MM099 melanoma cell cultured on collagen-coated stiff substrate, transfected with two different combinations of siRNA against DDR1 or DDR2 (siDDR1&2#1 and #2) or against YAP (siYAP#1 and #2). Cell nuclei were stained with Hoechst and counted using ImageJ software. Data are representative of 3 independent experiments. ***P < 0.001; ns, non-significant, Kruskal-Wallis analysis. **B** 1205Lu or MM099 cells treated as in (**A**) were seeded on stiff collagen for 24 h, and cell migration was recorded over 24 h and automatically tracked using ImageJ. For each condition, spider plots were created and cell displacement was analyzed using the ImageJ. **C** Quantification of cell displacement (*n* = 200 cells) from (**B**). Data represent median ± quartiles. Data are representative of 3 independent experiments. ***P < 0.001; ns non-significant, Kruskal-Wallis analysis. **D** Quantification of contractile forces applied by 1205Lu or MM099 cells transfected with two combinations of siRNA against DDR1 and DDR2 or YAP for 72 h and seeded on 4 kPa fluorescent bead-embedded collagen coated hydrogels for 24 h. Data are the mean ± SD (*n* = 10 cells/field from 5 random fields). Data are representative of 3 independent experiments. ***P < 0.001; ns non-significant, Kruskal-Wallis analysis. **E** *Top*, cell displacement analysis of 501Mel and MM074 cells transduced with a control (adCtrl) or a combination of DDR1 and DDR2 adenoviruses (adDDR1&2) and seeded on stiff substrate for 24 h. *Bottom*, quantification of cell displacement (*n* < 300 cells). Data represent median ± quartiles. Data are representative of 3 independent experiments. ***P < 0.001; ns non-significant, Kruskal-Wallis analysis. **F** 501Mel and MM074 cells transiently overexpressing DDR1 and DDR2 for 72 h were seeded on 4 kPa fluorescent bead-embedded collagen coated hydrogels for 24 h. Data are the mean ± SD (*n* = 10 cells/field from 5 random fields). Data are representative of 3 independent experiments. ***P < 0.001; ns non-significant, Kruskal-Wallis analysis. **G** 501Mel cells were transduced with a control (adCtrl) or a combination of DDR1 and DDR2 (adDDR1&2) adenoviruses prior transfection with control (siCtrl) or YAP (siYAP) siRNA. *Left*, displacement analysis of 501Mel cells seeded on stiff substrate for 24 h. *Right*, quantification of cell displacement (*n* < 300 cells). Data represent median ± quartiles. Data are representative of 3 independent experiments. ***P < 0.001; ns, non-significant, Kruskal-Wallis analysis.

We next sought to examine the consequences of modulating DDR-dependent mechanosignaling on proliferation and migration of melanoma cells. First, we used siRNA to knockdown DDR1 and DDR2 or YAP in 1205Lu or MM099 cells. Supplementary Fig. 4A shows the efficacy of YAP depletion by two different sequences of siRNA in both cell lines. Depletion of DDR or YAP reduced stiff ECM–induced proliferation (Fig. 5A) and migration (Fig. 5B, C) in dedifferentiated cells. Notably, YAP depletion decreased proliferation and migration without affecting cell viability (Supplementary Fig. 4B). Consistent with the decreased in MLC2 phosphorylation observed upon DDR knockdown (Fig. 4B), DDR or YAP-silenced cells generated weaker traction forces on their substrate (Fig. 5D). These results were confirmed by inhibiting DDR enzymatic activities with Imatinib or DDR1-in1 or impairing actomyosin cytoskeleton remodeling with the ROCK inhibitor Y27632 or the myosin II inhibitor Blebbistatin in 1205Lu and MM099 cells (Supplementary Fig. 4C–F). Conversely, ectopic expression of DDR1 and DDR2 triggered stiffness-dependent migration in differentiated cells (Fig. 5E), associated with stronger traction forces applied on stiff substrate (Fig. 5F). Notably, depleting YAP in those cells prevented stiffness-mediated migration (Fig. 5G). These data suggest that loss of rigidity sensing and signaling through DDR/actomyosin/YAP targeting suppresses mechanosensitive proliferation and migration in dedifferentiated melanoma cells.

### Targeting DDR or YAP sensitizes mechano-addicted melanoma cells to oncogenic BRAF pathway inhibition

**Fig. 6:**
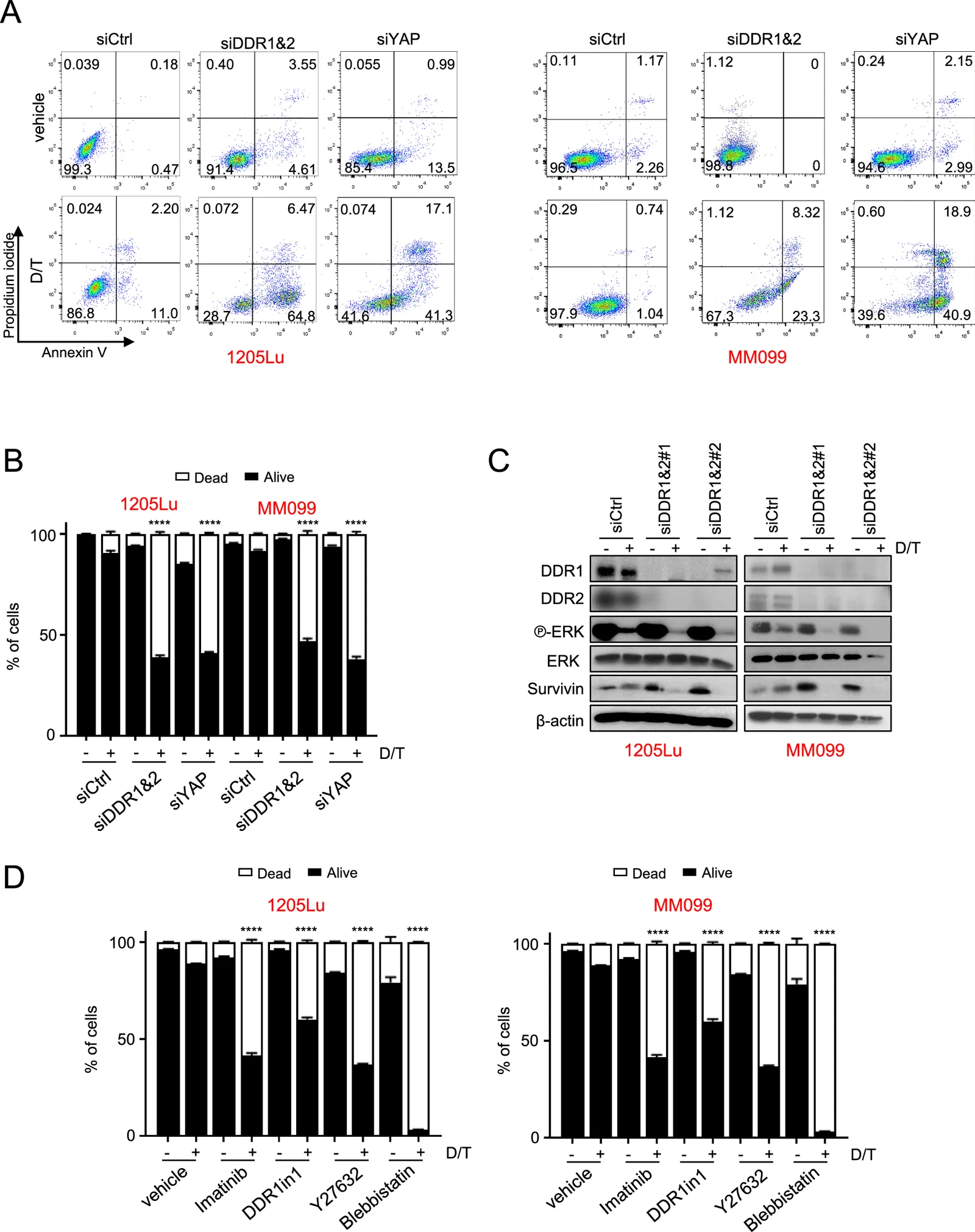
Targeting DDR or YAP sensitizes mechano-addicted melanoma cells to oncogenic BRAF pathway inhibition. Quantification of apoptosis in 1205Lu or MM099 cells seeded on stiff substrate and transfected with siRNA against DDR1 and DDR2 (siDDR1&2) or against YAP (siYAP) prior exposure to vehicle or 1 µM of Dabrafenib and 0.1 µM of Trametinib combotherapy (D/T) for 72 h. The percentage of dead cells was determined by flow cytometry analysis of annexin V-FITC/PI-stained cells. Dead cells (necrotic and apoptotic cells) are shown in the right lower quadrant, right and left upper quadrants. **B** Bar graph showing the percentage of dead (*white*) and live (*black*) cells from (**A**). Data are representative of 3 independent experiments. Data represent mean ± SD. ****P < 0.0001, D/T treatment compared to vehicle. Two-ANOVA analysis. **C** Immunoblot analysis of ERK1/2 phosphorylation and Survivin in lysates from 1205Lu and MM099 melanoma cells treated as in (**A**). β-Actin is used as loading control. **D** Bar graph showing the percentage of dead (*white*) and live (*black*) cells from 1205Lu and MM099 cells seeded on stiff substrate and treated with DDR inhibitors (Imatinib or DDR1in1) or with the ROCK inhibitor Y27632 or the myosin II inhibitor Blebbistatin in combination with 1 µM of Dabrafenib and 0.1 µM of Trametinib (D/T) for 72 h. The percentage of live and dead cells was determined by flow cytometry analysis of annexin V-FITC/PI-stained cells. Data are representative of 3 independent experiments. Data represent mean ± SD. ****P < 0.0001, D/T treatment compared to vehicle. Two-ANOVA analysis.

Given our previous findings that DDR contribute to ECM-mediated protection against BRAF-targeting therapy in BRAF-mutated melanoma cells [24], we investigated whether DDR are involved in ECM stiffness-induced resistance to the targeted drugs. We silenced DDR1 and DDR2 or YAP by siRNA in dedifferentiated cells and evaluated their responses to D/T combination treatment on stiff collagen substrate. Knockdown of DDR or YAP in 1205Lu or MM099 cells sensitized them to D/T treatment, as illustrated by the increased proportion of cells entering apoptosis (Fig. 6A, B). Accordingly, depletion of DDR1 and DDR2 in dedifferentiated cells led to a loss of the inhibitor apoptosis protein Survivin when cells were treated with D/T (Fig. 6C). Similar observations were obtained after pharmacological inhibition of DDR with Imatinib or DDR1-in1 as well as with Y27632 or Blebbistatin (Fig. 6D). Taken together, these results suggest that collagen receptors DDR contribute to drug resistant melanoma cell state observed in response to mechanical signals via the regulation of the actomyosin/YAP pathway.

## Discussion

ECM stiffening is a biophysical hallmark of solid tumors resulting from altered ECM deposition and collagen cross-linking. In cutaneous melanoma, recent observations have shown that high collagen expression correlates with poorer patient survival [28] and increased fibrillar ECM in patients with relapsed melanoma after failure of BRAF inhibitor treatment [20]. Consistently, an increase in the collagen fiber network associated with a pro-fibrotic response and tumor stiffening has been reported during adaptive response and resistance to BRAF-targeted therapy in several pre-clinical mouse models [19–22]. Despite the clinical relevance of a mechanical microenvironment in melanoma, little is known about how tumor matrix stiffness influences melanoma cell plasticity and phenotypic behavior.

In this study, we revealed that the transcriptional state of melanoma cells, rather than their mutational status is the main determinant of their mechanosensitivity, and that lineage dedifferentiation towards a mesenchymal-like phenotype confers on melanoma cells an increased sensitivity and addiction to ECM stiffness. Dedifferentiated cells showed increased proliferation, migration, and resistance to targeted therapy when exposed to stiff collagen, whereas melanocytic/transitory cells were less responsive to external stiffness. These findings support previously studies showing that ECM stiffening promotes the aggressiveness of several cancers [26, 29, 32, 33]. Further, we identified DDR1 and DDR2 as key collagen receptors contributing to the mechano-responsiveness of dedifferentiated melanoma cells. This is consistent with the observation that melanocytic cells show low DDR expression and lower reactivity on stiff ECM. In addition to different levels of DDR, the differential responses between melanocytic and dedifferentiated melanoma cells may also be due to variations in TGFβ signaling, that have been reported between these cell types [34]. Collagen stiffening was found to promote melanoma cell differentiation and MITF expression in the absence of TGFβ, whereas in the presence of TGFβ, it drives melanoma cell dedifferentiation and upregulates AXL expression [28]. However, our data show that ECM stiffness maintains phenotypic and cell-state transition markers in both melanocytic and mesenchymal dedifferentiated cells. This supports nicely the coexistence of melanocytic and dedifferentiated melanoma cells in collagen-rich tumor biopsies [28]. Maintaining phenotypic heterogeneity in collagen-dense regions could facilitate cooperation between different subpopulations of melanoma cells, a process known to promote invasion and metastasis [35–37].

Soft collagen or DDR inhibition was found to inhibit melanoma cell proliferation, suggesting that a soft tumor region or the loss of rigidity sensing may trigger non-proliferative/quiescent/dormant melanoma cells. Interestingly, tumor-derived type III collagen has been shown to sustain liver tumor dormancy by blocking DDR1/STAT1-mediated tumor cell proliferation [38]. Whether a similar mechanism operates in melanoma cells in a soft collagen environment remains to be determined. An intriguing question is why dedifferentiated cells cultured on soft collagen are more sensitive to targeted therapy if they are dormant. One possibility is that low DDR mechanical signaling may reduce mitochondrial metabolism, supported by the observation that reducing DDR1-dependent adhesion favors the transition from mesenchymal-like melanoma cells with high ATP levels to amoeboid invasive cells with low ATP levels [39].

Our previous research has shown that DDR1 and DDR2 levels increase during melanoma progression and are preferentially enriched in dedifferentiated melanoma cell subpopulations [24]. Targeting DDR1 in human melanoma cell lines decreases their proliferation, invasion, and survival [40, 41], while depleting DDR2 in murine melanoma cell lines reduces their metastatic potential [42]. Further, DDR2 inhibition was recently shown to decrease AXL expression and counteract acquired resistance in BRAFi-resistant melanoma cells [43]. Consistent with our findings that both receptors are required to mediate fibroblast-derived matrix protection against MAP kinase inhibitors [24], we show that DDR1 and DDR2 are required to transduce ECM mechanical signals and drug resistance in dedifferentiated cells. Moreover, forced expression of DDR in melanocytic/transitory cells is sufficient to decrease expression of the differentiation marker MITF and enhances their response to stiff ECM. Our results are consistent with studies showing that DDR1 senses matrix stiffness and regulates vascular smooth muscle cells (VSMCs) through RhoA GTPase activation and YAP signaling, creating a feedback loop that further enhances DDR expression [5, 44]. Our data indicate that inhibition of actomyosin remodeling by blebbistatin prevents DDR-mediated YAP activation by stiff ECM. However, whether these events involve a direct DDR-myosin II interaction as described for DDR1 during cell migration and collagen mechanical remodeling [31, 45] remains to be determined.

β1-integrins, another class of collagen receptors, play a critical role in mechanosensing [29, 32] and, in melanoma, contribute to fibronectin-mediated resistance to BRAF inhibition [18, 20]. While β1-integrin expression is elevated in dedifferentiated cells, its modulation by collagen stiffening is variable across cell lines, unlike DDR1 and DDR2, which show a more consistent response. However, the observation that stiff collagen increases focal adhesion formation and number suggests a potential role for β1-integrin downstream signaling in supporting the mechanosensitivity of dedifferentiated melanoma cells. Given this, the interaction between DDR and β1-integrin could confer these properties, as DDR1 has been shown to regulate integrin interaction with fibrillar collagen in other cell types [46]. Similarly, DDR2 controls breast tumor stiffness and metastasis by regulating integrin-mediated mechanotransduction in cancer-associated fibroblasts [30]. Whether β1-integrins contribute to DDR-mediated mechanotransduction in the melanoma cell context requires further experimentations. Along the same lines, it would be interesting to examine the relationship between the DDR/1/2-YAP axis and cadherins, other important mechanosensors in melanoma phenotypic plasticity and progression [47].

Our findings show that stiff collagen enhances therapy resistance by activating DDR receptors and YAP-dependent mechanotransduction pathways. Inhibition of DDR or YAP as well as ROCK-myosin II signaling in dedifferentiated melanoma cells dampens the protective effect conferred by stiff collagen, thereby sensitizing these cells to combined BRAF and MEK inhibition. AXL has been shown to play a major role in resistance to targeted therapy in dedifferentiated melanoma cells [9, 48]. Thus, the reduction of AXL on soft ECM is likely to contribute to the increased drug sensitivity observed in dedifferentiated cells in this setting. We envision that collagen stiffening, by maintaining the dedifferentiated mesenchymal phenotype of melanoma cells and activating mechanotransduction pathways, may also influence melanoma response to immunotherapies. Indeed, immune checkpoint inhibitor resistance is linked to melanoma subtypes with mesenchymal and YAP-dependent mechanical phenotypes [13, 49–51]. Consistently, upregulation of the EMT transcription factor ZEB1 on stiff collagen that we observed could favor melanoma immune evasion and immunotherapy efficacy [52]. Notably, reversing tumor stiffening by inhibiting collagen cross-linking has been shown to improve T cell migration and the efficacy of anti-PD1 treatment [53], suggesting that softening tumor tissue could improve the efficacy of immunotherapies in melanoma patients. Collectively, these observations support the notion that therapeutic strategies aimed at disrupting DDR-collagen interactions [24, 54] or inhibiting tumor stiffness-induced mechanotransduction pathways [19, 55] may prevent melanoma phenotypic adaptation and resistance to current melanoma therapies.

In conclusion, targeting mechanical stimuli from the ECM holds significant potential for enhancing the efficacy of therapies in melanoma, as well as other collagen-rich and stiff tumors. A deeper understanding of the mechanisms by which ECM stiffness and mechanotransduction contribute to tumor cell behavior and plasticity may pave the way for novel strategies to combat resistant and metastatic cancer cell phenotypes. In this work, we describe differential mechanical responsiveness between opposite tumor cell phenotypes (proliferative and invasive) and reveal that mechanical addiction driven by DDR collagen receptors may represent a novel therapeutic vulnerability of the dedifferentiated mesenchymal-like cell state in melanoma.

## Materials and methods

### Cells and reagents

Melanoma cell lines 501Mel (RRID:CVCL_4633) were obtained as described before [56, 57]. 1205Lu cells (RRID:CVCL_5239) were from Rockland (USA). Short-term melanoma cultures were described previously [24, 34]. Cell lines were cultured in Dulbecco’s modified Eagle Medium (DMEM), 7% FBS (Hyclone) and 1% Penicillin/Streptomycin (Gibco). Short-term cells were cultured in Ham’s F10 nutrient mix (Invitrogen), 10% FBS (Hyclone), 7% Glutamax (Gibco) and 1% penicillin/streptomycin (Invitrogen). To guarantee cells authenticity, cells were expanded and frozen at low passage after their receipt from original stocks and used for a limited number of passages after thawing. All cell lines were routinely tested for the absence of mycoplasma by PCR. Culture reagents were purchased from Thermo Fisher Scientific. Dabrafenib, Trametinib, Y27632, Blebbistatin and DDR1in1 were from Selleckchem. Imatinib mesylate was from Enzo Life Science. Adenoviruses expressing DDR1 and DDR2 were from Applied Biological Materials and were amplified as described [57].

### Preparation of substrates of different rigidity

#### Polyacrylamide hydrogels

Hydrogels were generated from a mixture of 40% acrylamide (Fisher Bioreagents) and 2% bisacrylamide (Fisher Bioreagents) to obtain different rigidities. This mixture was cast between two glass slides. After 10 min of polymerization at room temperature and treatment with sulfo-SANPAH (Thermo Fisher), the hydrogels were coated for 45 min at 37 °C with bovine type I collagen (50 µg/ml, ThermoFisher) and then sterilized under UV for 30 min. After 45 min equilibration in DMEM medium, cells can be seeded on it [58].

#### Collagen gels

Soft collagen gels were made by mixing type I collagen (2.5 mg/ml) in PBS and NaOH (0.1 N). The mixture was added to the bottom of tissue culture multiwell plates and incubated for 1 h at 37 °C. Stiff substrates were generated by coating multiwell plates with type I collagen at 50 µg/ml for 1 h at 37 °C [59].

### Cell proliferation

Cells were plated in 48-well dishes at 10^4^ cells in triplicate on soft or stiff substrate. For siRNA experiments, cells were transfected for 72 h then detached using 0.05% trypsin and replated in collagen coated plates. Proliferation was followed for 72 h by counting the nuclei of NucLight Red-expressing cells using the IncuCyte ZOOM™ system according to the manufacturer’s instructions (Essen BioScience). For siRNA experiments, cells were transfected for 72 h then detached using 0.05% trypsin, replated in collagen coated plates and monitored as described above. Alternatively, cells were fixed with paraformaldehyde and labeled with Ki67 and Hoechst (33342, Invitrogen). Cells were imaged using a fluorescence microscope (Leica DM5500B, 10X magnification).

### Flow cytometry

#### Cell cycle analysis

Cells were plated on soft or stiff substrate and treated or not with the indicated drugs or transfected with siRNA for 72 h. Cells were detached with Collagenase I (Gibco), washed and fixed in ice-cold ethanol 70% for at least 24 h. Cells were stained for 15 min at RT with 40 μg/mL PI (Sigma) in FACS Buffer (1X PBS + 0.5% BSA + 1 mM EDTA) supplemented with 100 μg/mL RNAse A (Sigma). Cell cycle profiles were collected on FACS Canto II cytometer (Becton Dickinson). FACS data were analyzed using FlowJo software.

#### Apoptosis analysis

Cells were plated on soft or stiff substrate and treated with the indicated drugs or transfected with siRNA for 72 h. Cells were then detached with Collagenase I (Gibco), washed in Annexin-V binding buffer (Miltenyi Biotec) and stained with Annexin-V-FITC and PI for 15 min at RT in the dark, according to the manufacturer’s instructions (Miltenyi Biotec). Cell death was analyzed on FACS Canto II cytometer (Becton Dickinson). FACS data were analyzed using FlowJo software.

### Time-lapse migration assays

Cells were plated in 24-well dishes at 2 × 10^4^ cells on soft or stiff substrate for 24 h. For siRNA transfection, treatments with indicated drugs or viral transduction, cells were transfected, treated or virally transduced for 72 h then detached using 0.05% trypsin and replated in collagen coated 24-well plates. Cells were stained with Hoechst (Thermofisher, 125 ng/ml) for 5 min, then washed and their migration was assessed by time-lapse live imaging for 24 h with one image taken every 10 min, using the inverted widefield microscope (Leica TIRF, 5X) under 37 °C and 5% CO_2_. Cell displacement was analyzed using the semi-automated plugin TrackMate [60] of Fiji software (ImageJ).

### Invasion assays

Cells were seeded for 7 days on soft or stiff substrate and starved for 24 h then dissociated with 0.05% trypsin and counted. 1.5 × 10^5^ cells were loaded in the upper compartment of the transwell (8 µm, Corning), coated with Matrigel (1 mg/ml) and FBS containing-medium was added in the lower compartment of the transwell. After 24 h, migrating cells were fixed, and nuclei were stained with Hoechst. Pictures were taken with the inverted widefield microscope (Leica DM5500B, 10X). Quantification was done with Fiji software (ImageJ).

### RNAi studies and adenoviral transduction

siRNA used in this study are listed in Supplementary Table 2. 50 nM siRNA were transfected using Lipofectamine RNAi MAX (ThermoFisher Scientific), following manufacturer’s instructions for 72 h.

For DDR1/2 overexpression, cells were transduced with DDR1- and DDR2-expressing adenoviruses (1 µL viral stock per well, 6-well plate) for 72 h. Transduction conditions were selected based on preliminary optimization to achieve robust expression with minimal cytotoxicity.

### Immunoblot analysis and antibodies

Cells were collected 72 h after being seeded on soft vs stiff substrate or 72 h after siRNA transfection. Cell lysates were subjected to immunoblot analysis as described before [57] using antibodies listed in Supplementary Table 3.

### Real-time quantitative PCR

Gene expression levels were determined by RT-qPCR as described [56]. Data were presented as relative gene expression according to the ΔΔCt method. Heatmaps depicting fold changes of gene expression were prepared using MeV software. Primer sequences are listed in Supplementary Table 4.

### Traction force microscopy (TFM)

Cells were plated for 48 h on collagen-coated polyacrylamide hydrogels with shear modulus of 0.2 kPa or 4 kPa containing red, fluorescent beads (SoftTrac, Cell Guidance Systems). For siRNA transfection, treatments with indicated drugs or viral transduction, cells were transfected, treated or virally transduced for 72 h then detached using 0.05% trypsin and replated on 4 kPa SoftTrac. Images were acquired before and after cell removal using a fluorescence microscope (Leica TIRF, 10X magnification). Tractions exerted by cells were estimated by measuring beads displacement fields, computing corresponding traction fields using Fourier transformation and calculating root-mean-square traction using the particle image velocity (PIV) plugin on ImageJ [61]. The same procedure was performed on a cell-free region to measure baseline noise.

### Immunofluorescence analysis

After 72 h of growth on soft or stiff substrates, after siRNA transfection, inhibitor treatments or viral transduction, cells were rinsed, fixed in 4% PFA, incubated in blocking buffer (0.3% triton 5% Goat Serum in PBS) for 1 h and then with the indicated primary antibodies overnight at 4 °C. Following incubation with Alexa Fluor-conjugated secondary antibodies (1:200) and nucleus staining with Hoechst, coverslips and hydrogels were mounted in Prolong antifade (Thermo Fisher Scientific). F-actin was stained with Alexa Fluor594 or Alexa Fluor488 phalloidin (1:200, Thermo Fisher Scientific). Images were captured on a widefield microscope (Leica DM5500B, 40X magnification). Quantifications of cell area, YAP nuclear localization and the number and size of focal adhesions were performed with Image J software.

YAP nuclear localization was quantified in Fiji by segmenting nuclei with DAPI and the cytoplasm with F-actin, then measuring YAP fluorescence intensity in each region. The nuclear-to-cytoplasmic YAP ratio was calculated for each cell to assess localization.

Focal adhesion number and size were quantified in Fiji by thresholding the phospho-tyrosine signal to generate a binary mask, then using Analyze Particles to measure focal adhesion count and area. All images were processed using identical threshold and analysis parameters to ensure consistency.

### Statistical analysis

Unless otherwise stated, all experiments were repeated at least three times and representative data/images are shown. Statistical data analysis was performed using GraphPad Prism software. Unpaired two-tailed Mann-Whitney test was used for statistical comparisons between two groups and Kruskal-Wallis test with Dunn post-tests or two-way analysis of variance test with Bonferroni post-tests to compare three or more groups. Histogram plots and curves represent mean ± SD and Violin plots represent median with interquartile range. *P* ≤ 0.05 were considered statistically significant.

## Supplementary information

Supplementary material legends

Supplementary Figure 1

Supplementary Figure 2

Supplementary Figure 3

Supplementary Figure 4

Supplementary Table 1

Supplementary Table 2 to 4

## Data Availability

Additional data are available in the supplemental information. The data that support the findings of this study are available from the corresponding author upon reasonable request.
