# Supplementary material for: Extracellular matrix stiffness determines the phenotypic behavior of dedifferentiated melanoma cells through a DDR1/2-dependent YAP mechanotransduction pathway
Source: Oncogenesis. 2026 Jun 13;15(1):46. doi: 10.1038/s41389-026-00636-y (PMC13490450; doi:10.1038/s41389-026-00636-y)
Supplement: Supplementary file 1 [file 41389_2026_636_MOESM1_ESM.pdf]

**Supplementary material legends**

**Supplementary Table 1.** Phenotypic state and mutation status of the different melanoma cell lines and short-term patient derived melanoma cells used in the study.

**Supplementary Table 2.** List of siRNAs used in the study.

**Supplementary Table 3.** List of antibodies used in this study. WB : Western Blotting, IF : Immunofluorescence.

**Supplementary Table 4.** List of primers used for real-time qPCR in this study.

**Supplementary Figure 1:** (A) Representative images of the morphology of short-term patient derived cells presenting either a melanocytic/transitory (in blue) or a dedifferentiated (in red) phenotype cultured for 72h on collagen-coated soft or stiff substrate. F-Actin was stained with phalloidin (green) and nuclei with Hoechst (blue). Scale bar, 50µm. (B) Immunofluorescence analysis of short-term patient derived cells focal adhesions cultured as in (A). Focal adhesions were stained with anti-Phospho-Tyrosine antibody (green), F-Actin with Phalloidin (Red) and nuclei with Hoechst (Blue). Insets show higher magnification views of delineated zones. Scale bar, 20µm. (C) Effect of collagen stiffening on YAP nuclear translocation assessed by immunofluorescence in short-term patient derived cells focal adhesions cultured as in (A). Insets show F-Actin in green and nuclei in blue. Scale bar, 15µm. (D-E) qPCR analysis of YAP target genes expression in short-term patient derived cells (D) or in melanoma cell lines (E) cultured as in (A) for 72h. Data are normalized to the expression in cells plated on soft substrate. Data are represented as mean ± SD from a technical triplicate representative of 3 independent experiments. ***, P<0.001; ns, non-significant, two-way ANOVA analysis. (F) Expression of *YAP1*, *CYR61*, *AXL* and *MITF* in dedifferentiated (D) and melanocytic (M) melanoma cells. Data were obtained from the Graeber's lab Melanoma Gene Expression database https://systems.crump.ucla.edu/dediff/index.php [17].

**Supplementary Figure 2:** (A) *Top*, Proliferation of short-term patient derived melanoma cells cultured for 72h on soft or stiff collagen-coated substrates, assessed by Ki67 staining (green). Scale bar, 150µm. *Bottom*, Bar graph representing the % of Ki67 positive cells per field. 6-7 fields containing each 20-100 cells were analysed. Data are representative of 3 independent experiments. Data represent mean ± SD. ***, P<0.001; **, P<0.01; ns, non-significant, Kruskal-Wallis analysis. (B) *Top,* Proliferation of 1205Lu or 501Mel cells plated for 72h on soft or stiff collagen substrates assessed by Ki67 staining (green). Scale bar, 150µm. *Bottom*, Bar graph representing the % of Ki67 positive cells per field. 5 fields containing each 20-100 cells were

analysed. Data are representative of 3 independent experiments. Data represent mean ± SD. ***, $P<0.001$; *, $P<0.05$, Kruskal-Wallis analysis. (C) Immunoblot of indicated markers of phenotype switching on lysates from cell lines or short-term patient derived cells cultured for 72h on soft or stiff collagen-coated substrates. HSP90 is used as loading control. (D) Cell cycle distribution of melanoma short-term patient derived cells cultured for 72h on soft or stiff collagen-coated substrates, analysed by flow cytometry. (E) Cell cycle distribution of 1205Lu, MM099 or 501Mel cells seeded on soft or stiff substrate collagen-coated and treated or not (vehicle) for 72h with 1µM of Dabrafenib and 0.1µM of Trametinib (D/T).

**Supplementary Figure 3:** (A-C) 1205Lu or MM099 dedifferentiated melanoma cells seeded on stiff collagen substrate were treated for 72h with DDR inhibitors Imatinib (7µM) or DDR1in1 (1µM). (A) Immunoblot analysis of activated (phosphorylated) and total forms of DDR1, DDR2 and MLC2 on cell lysates from 1205Lu or MM099 treated as above. β-Actin is used as loading control. (B) *Left,* Immunofluorescence analysis of focal adhesions of 1205Lu or MM099 cells described above. Focal adhesions were stained with anti-Phospho-Tyrosine antibody (green), F-Actin with Phalloidin (red) and nuclei with Hoechst (blue). Insets show higher magnification views of zones delineated by white square. Scale bar, 20µm. *Right-top*, Quantification of focal adhesions per cell (*top*) or focal adhesion area (*bottom*) of the indicated melanoma cell lines cultured as above. Data are representative of 3 independent experiments. *top*, each dot represents a field comprising 3-10 cells. Data are represented as scatter plot with mean ± SD from 10 different fields. ***, $P<0.001$; **, $P<0.01$, Kruskal-Wallis analysis. bottom, bar graph represents the mean ± SD ($n > 150$ cells) of triplicate experiments. ***, $P<0.001$; **, $P<0.01$; *, $P<0.05$, Kruskal-Wallis analysis. (C) *Left*, Immunofluorescence analysis of YAP (red) of 1205Lu or MM099 cells described above. Insets show nuclei staining by Hoechst. Scale bar, 15µm. *Right*, quantification of the nucleocytoplasmic distribution of YAP ($n \geq 30$ cells per condition). Data represented mean ± SD of 3 independent experiments. ***, $P<0.001$; **, $P<0.01$; *, $P<0.05$, Kruskal-Wallis analysis. (D) qPCR analysis of YAP target genes expression in 1205Lu or MM099 Dedifferentiated melanoma cells seeded on high stiffness substrate and either transfected with siRNA against DDR1 and DDR2 (*top*) or treated with DDR inhibitors (*bottom*) for 72h. Data are normalized to the expression in cells transfected with siCtrl or treated with vehicle, respectively. Data are represented as mean ± SD from a technical triplicate representative of 3 independent experiments. ***, $P<0.001$; ns, non-significant, two-way ANOVA analysis. (E) Spearman's correlation analysis of DDR1 and YAP (*left*) or DDR2 and YAP (*right*) mRNA expression from data of skin melanoma extracted from the TCGA. (F) Kaplan–Meier survival curves for melanoma patients stratified into low- and high-risk groups based on a multivariable Cox proportional hazards model including YAP, DDR1, and DDR2 expression. The risk score was dichotomized at its median. P-values were calculated using

the log-rank test. (G) Immunoblot analysis of indicated markers of lysates from 501Mel and MM074 cells cultured on stiff substrate and transduced for 72h with control adenoviruses (adCtrl) or adenoviruses expressing DDR1 and DDR2 (adDDR1&2). β-Actin is used as loading control. (H) *Left,* Representative images of 501Mel and MM074 cells cultured on stiff substrate transduced for 72h with adenoviruses expressing DDR1 and DDR2. F-Actin was stained with phalloidin (green) and nuclei with Hoechst (blue). Scale bar, 50µm. *Right*, quantification of cell area represented as scatter plot with mean ± SD (n > 150 cells). Data are representative of 3 independent experiments. ***, P<0.001, Kruskal-Wallis analysis. (I) qPCR analysis of YAP target genes expression in 501Mel and MM074 melanocytic cells transduced with adenoviruses expressing DDR1 and DDR2 and treated with Blebbistatin (40µM) for 72h on stiff substrate. Data are normalized to the expression in cells transduced with control adenovirus (adCtrl) and treated with vehicle. Data are represented as mean ± SD from a technical triplicate representative of 3 independent experiments. ***, P<0.001; **, P<0.01; *, P<0.05, two-way ANOVA analysis. (J) Proliferation analysis of 501Mel and MM74 melanoma cell cultured on collagen-coated stiff substrate, transduced for 72h with adDDR1&2 or adCtrl. Cell nuclei were stained with Hoechst and counted using ImageJ software. Data are representative of 3 independent experiments. ns, non-significant, Mann-Whitney analysis.

**Supplementary Figure 4:** (A) Immunoblot analysis of YAP expression from 1205Lu and MM099 transfected with siCTRL, siYAP#1 or siYAP#2. β-Actin is used as loading control. (B) Analysis of cell death in 1205Lu transfected as in (A) for 72h. The percentage of dead cells was determined by flow cytometry analysis of annexin V-FITC/PI-stained cells. Dead cells (necrotic and apoptotic cells) are shown in the right lower quadrant, right and left upper quadrants. Bar graph shows the percentage of dead (white) and live (black) cells. (C) Proliferation analysis of 1205Lu and MM099 melanoma cell cultured on collagen-coated stiff substrate, treated for 72h with DDR inhibitor Imatinib (7µM) or DDR1in1 (1µM), ROCK inhibitor Y27632 (40µM) or myosin II inhibitor Blebbistatin (40µM). Cell nuclei were stained with Hoechst and counted using ImageJ software. Data are representative of 3 independent experiments. ***, P<0.001; ns, non-significant, Kruskal-Wallis analysis. (D) *Top*, Cell displacement analysis of 1205Lu and MM099 cells treated with as in (C) and seeded on stiff substrate for 24h. *Bottom*, Quantification of cell displacement (n = 200 cells). Data represent median ± quartiles. Data are representative of 3 independent experiments. ***, P<0.001; ns, non-significant, Kruskal-Wallis analysis. (E) Quantification of contractile forces applied by 1205Lu or MM099 cells treated as in (C) and seeded on 4kPa fluorescent beads-embedded collagen coated hydrogels for 24h. Data are the mean ± SD (n = 10 cells/field from 5 random fields). Data are representative of 3 independent experiments. ***, P<0.001; ns, non-significant, Kruskal-Wallis analysis. (F) Immunoblot analysis of activated (*phosphorylated*) and

total forms of MLC2 on cell lysates from 1205Lu and MM099 melanoma cells treated for 72h with ROCK inhibitor Y27632 (40μM) or myosin II inhibitor Blebbistatin (40μM). β-Actin is used as loading control.
