# Supplementary material for: Extracellular matrix stiffness determines the phenotypic behavior of dedifferentiated melanoma cells through a DDR1/2-dependent YAP mechanotransduction pathway
Source: Oncogenesis. 2026 Jun 13;15(1):46. doi: 10.1038/s41389-026-00636-y (PMC13490450; doi:10.1038/s41389-026-00636-y)

Supplementary Figure 4

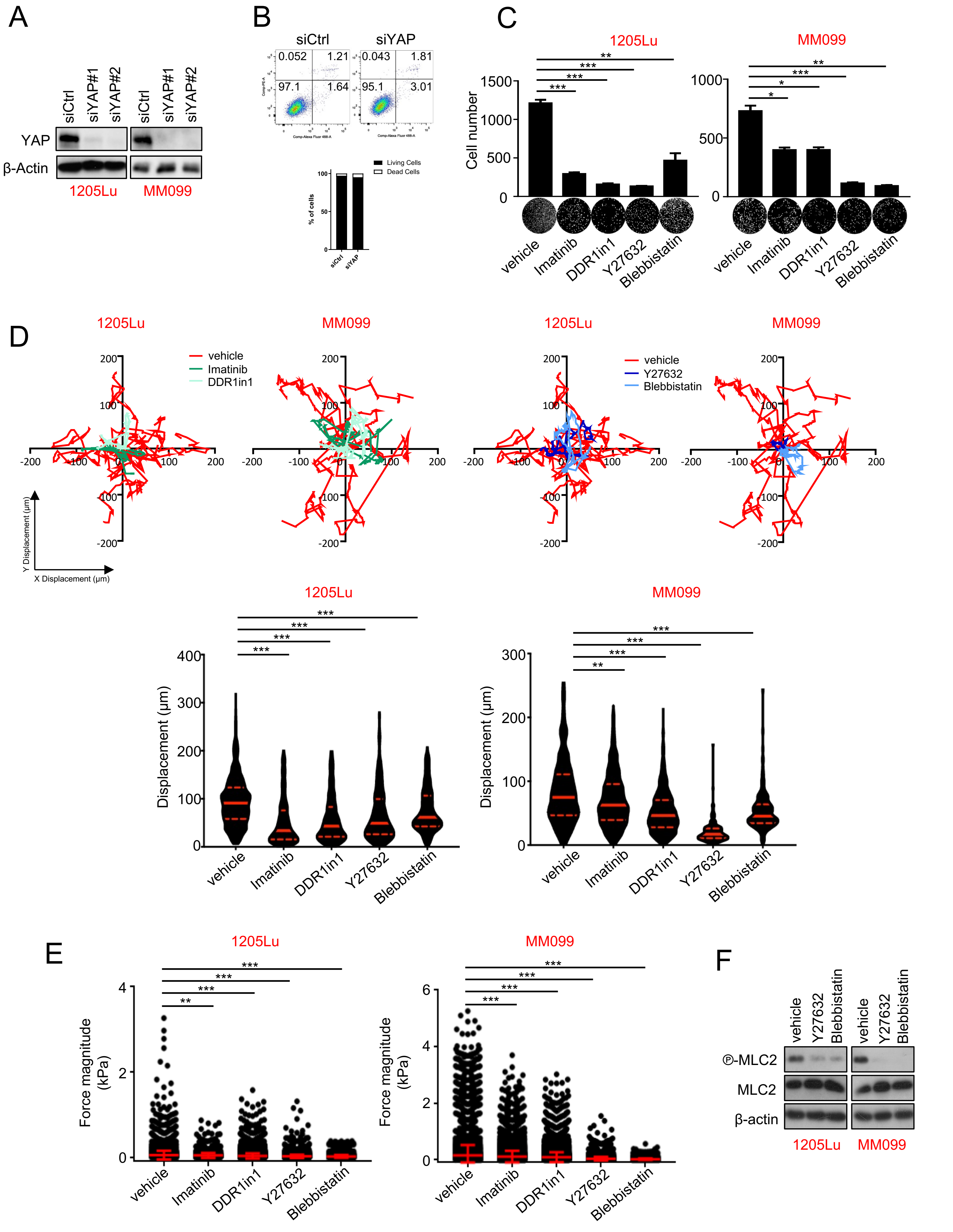

Supplement: Supplementary file 5 [file 41389_2026_636_MOESM5_ESM.pdf]
