# Supplementary material for: Extracellular matrix stiffness determines the phenotypic behavior of dedifferentiated melanoma cells through a DDR1/2-dependent YAP mechanotransduction pathway
Source: Oncogenesis. 2026 Jun 13;15(1):46. doi: 10.1038/s41389-026-00636-y (PMC13490450; doi:10.1038/s41389-026-00636-y)
Supplement: Supplementary file 6 [file 41389_2026_636_MOESM6_ESM.pdf]

Supplementary Table 1

	Mutation status	Phenotypic state
Cell line		
1205Lu	BRAFV600E	Dedifferentiated
501Mel	BRAFV600E	Melanocytic
Short-term patient-derived cell		
MM029	BRAFV600E	Dedifferentiated
MM099	BRAFV600E	Dedifferentiated
MM074	BRAFV600E	Intermediate/Transitory
MM034	BRAFV600E	Melanocytic
MM165	NRASQ61L	Dedifferentiated
MM047	NRASQ61L	Dedifferentiated
MM057	NRASQ61L	Intermediate/Transitory
MM001	NRASQ61L	Melanocytic
