# Supplementary material for: Extracellular matrix stiffness determines the phenotypic behavior of dedifferentiated melanoma cells through a DDR1/2-dependent YAP mechanotransduction pathway
Source: Oncogenesis. 2026 Jun 13;15(1):46. doi: 10.1038/s41389-026-00636-y (PMC13490450; doi:10.1038/s41389-026-00636-y)
Supplement: Supplementary file 7 [file 41389_2026_636_MOESM7_ESM.pdf]

Supplementary Table 2

Name	Target RNA	Reference	Manufacturer
siCtrl	Luciferase	12935146	Thermo Fisher Scientific
siYAP#1	YAP1	#HSS115942	Thermo Fisher Scientific
siYAP#2	YAP1	#HSS115944	Thermo Fisher Scientific
siDDR1&2#1	DDR1 & DDR2	#HSS187878 & #HSS107350	Thermo Fisher Scientific
siDDR1&2#2	DDR1 & DDR2	#HSS187879 & #HSS107352	Thermo Fisher Scientific

Supplementary Table 3

Name	Manufacturer, Ref, RRID	Dilution	Application
AXL	Cell Signaling Technology Cat# 4566, RRID: AB_2062563	1:1000	WB
β-Actin	Cell Signaling Technology Cat# 3700, RRID: AB_2242334	1:5000	WB
β1-Integrin	Sigma Cat# MAB1965, RRID: AB_2128061	1:1000	WB
Survivin	Cell Signaling Technology Cat# 2808, RRID:AB_2063948	1:1000	WB
CTGF	Santa Cruz Biotechnology Cat# sc-365970, RRID: AB_10917259	1:500	WB
CYR61	Santa Cruz Biotechnology Cat# sc-374129, RRID:	1:500	WB
DDR1	Cell Signaling Technology Cat# 5883, RRID: AB_10947399	1:1000	WB
Phospho-DDR1 (Tyr792)	Cell Signaling Technology Cat# 11994, RRID: AB_2797793	1:1000<br>1:200	WB<br>IF
DDR2	Cell Signaling Technology Cat# 12133, RRID: AB_2797825	1:1000	WB
Phospho-DDR1 (Tyr796)/DDR2 (Tyr740)	R&D Systems Cat# MAB25382,	1:1000<br>1:200	WB<br>IF
E2F1	Cell Signaling Technology Cat# 3742, RRID: AB_2096936	1:1000	WB
ERK2	Santa Cruz Biotechnology Cat# sc-1647, RRID: AB_627547	1:500	WB
Phospho-ERK1/2 (Thr202/Tyr204)	Cell Signaling Technology Cat# 9101, RRID: AB_331646	1:1000	WB
HSP60	Santa Cruz Biotechnology Cat# sc-59567, RRID: AB_783870	1:500	WB
HSP90	Santa Cruz Biotechnology Cat# sc-13119, RRID: AB_675659	1:1000	WB
Ki67	Abcam Cat# ab-16667, RRID: AB_302459	1:200	IF
MITF	Sigma Cat# HPA003259, RRID: AB_1079381	1:1000	WB
MLC2	Cell Signaling Technology Cat# 8505, RRID: AB_2728760	1:1000	WB

Phospho-MLC2 (Ser19)	Cell Signaling Technology Cat# 3671, RRID: AB_330248	1:1000	WB
P27Kip1	Cell Signaling Technology Cat# 3686, RRID: AB_2077850	1:1000	WB
Rb	Cell Signaling Technology Cat# 9309, RRID: AB_823629	1:1000	WB
Phospho-Rb (Ser807/Ser811)	Cell Signaling Technology Cat# 9308, RRID: AB_331472	1:1000	WB
Phospho-Tyrosine (Tyr-100)	Cell Signaling Technology Cat# 9411, RRID: AB_331228	1:800	IF
YAP	Cell Signaling Technology Cat# 14074, RRID: AB_2650491	1:1000<br>1:100	WB<br>IF
ZEB1	Santa Cruz Biotechnology Cat# sc-25388, RRID: AB_2217979	1:500	WB
Anti-mouse IgG-HRP	Cell Signaling Technology Cat# 7076, RRID: AB_330924	1:2000	WB
Anti-rabbit IgG-HRP	Cell Signaling Technology Cat# 7074, RRID: AB_2099233	1:2000	WB
Goat anti-rabbit AF488	ThermoFischer Cat# A11034, RRID: AB_2576217	1:200	IF
Goat anti-mouse AF488	ThermoFischer Cat# A11029, RRID: AB_2534088	1:200	IF
Goat anti-rabbit AF594	ThermoFischer Cat# A11012, RRID: AB_141359	1:200	IF
Goat anti-mouse AF594	ThermoFischer Cat# A11005, RRID: AB_141372	1:200	IF

Supplementary Table 4

Name	Forward sequence (5'->3')	Reverse sequence (5'->3')
AMOTL2	GCGACTGTCAGAACAACTGC	GCACCTTTAACCTGCTTTCCA
AXL	GTGGGCAACCCAGGGAATATC	GTACTGTCCCGTGTCGGAAAG
CTGF	ACCGACTGGAAGACACGTTTG	CCAGGTCAGCTTCGCAAGG
CYR61	TGAAGCGGCTCCCTGTTTT	CGGGTTTCTTTCACAAGGCG
CDH2	CCACGCCGAGCCCCAGTAT	GGCCCCCAGTCGTTCAGGTAAT
CTNNB1	CCGCAAATCATGCACCTTT	ATTTCTTCCATGCGGACCC
DDR1	ATGGAGCAACCACAGCTTCTC	CTCAGCCGGTCAAACTCAAACT
DDR2	AGTCAGTGGTCAGAGTCCACAGC	CAGGGCACCAGGCTCCATC
ERBB3	GGTGATGGGGAACCTTGAGAT	CTGTCACTTCTCGAATCCACTG
HPRT	AGATGGTCAAGGTCGCAAGCT	AGTCAAGGGCATATCCTACAACAAAC
MITF	CATTGTTATGCTGGAAATGCTAGAA	GGCTTGCTGTATGTGGTACTTGG
MLANA	GCTCACTTCATCTATGGTTACCC	GACTCCCAGGATCACTGTCAG
NGFR	CCTACGGCTACTACCAGGATG	CACACGGTGTTCTGCTTGT
RPL32	CCTTGTGAAGCCCAAGATCG	TGCCGGATGAACTTCTTGGT
Serpine1	CCGCCTCTTCCACAAAATCA	CAGTTCCAGGATGTCGTAGTAATGG
SOX9	CCCAACGCCATCTTCAAGG	GAGACCGTCCCTGCTGTGTT
SOX10	CCTTCATGGTGTGGGCTC	CGCTTGTCACTTTCGTTCAG
Wnt5a	CAATGTCTTCCAAGTTCTTCCTAGTG	CATACCTAGCGACCACCAAGAAT
YAP1	GCTACAGTGTCCCTCGAACC	CCGGTGCATGTGTCTCCTTA
ZEB1	AGCGCTTCTCACACTCTGGGTCTT	CTGCCTCTTCCTGCTCTGTGC
ZEB2	GCGCAGTGACACAGCCATTATTTA	CTTGTAGCCCCGGTCGCAGTA
